# Commentary: Consensus definition of misophonia

**DOI:** 10.3389/fnins.2022.1077097

**Published:** 2023-01-04

**Authors:** Hashir Aazh

**Affiliations:** ^1^Audiology Department, Royal Surrey NHS Foundation Trust, Guildford, United Kingdom; ^2^Department of Communication Sciences and Disorders, Florida Atlantic University, Boca Raton, FL, United States; ^3^Faculty of Engineering and Physical Sciences (FEPS), University of Surrey, Guildford, United Kingdom; ^4^Hashir International Specialist Clinics and Research Institute for Misophonia, Tinnitus and Hyperacusis, Guildford, United Kingdom

**Keywords:** misophonia, hyperacusis, consensus, hearing disorders, audiology

There are different types of sound intolerance ranging from misophonia and hyperacusis to noise sensitivity (Jastreboff and Jastreboff, 2002; Aazh et al., 2014, 2018; Henry et al., 2022). The focus of this paper is on misophonia. There are conflicting results about the prevalence of misophonia ranging from 5 to 49.1% (Wu et al., 2014; Siepsiak et al., 2020; Naylor et al., 2021; Jakubovski et al., 2022). There are also debates about the mechanism of misophonia in fields of psychiatry, neurology, and neuroscience (Jastreboff and Jastreboff, 2003; Cavanna and Seri, 2015; Erfanian et al., 2019). For example, some researchers classify misophonia as a psychiatric illness (Schroder et al., 2013) and others as a neurodevelopmental disorder (Cavanna and Seri, 2015). In the field of audiology, misophonia is often considered as a subtype of hyperacusis rather than a distinct disorder (Tyler et al., 2014). Therefore, most research studies in the field of audiology have not distinguished misophonia from hyperacusis (Fackrell et al., 2015; Sheldrake et al., 2015; Zaugg et al., 2016; Aazh et al., 2017). Tyler et al. (2014) described four categories of hyperacusis comprising (1) loudness hyperacusis, (2) fear hyperacusis, (3) pain hyperacusis, and (4) annoyance hyperacusis which overlaps with misophonia.

Recently, the Milken Institute Center for Strategic Philanthropy through a grant from The REAM Foundation's Misophonia Research Fund, assembled a group of scientific researchers and clinicians with expertise in misophonia and related topics in order to develop a consensus definition for misophonia (Swedo et al., 2022). To raise awareness about this consensus definition among audiologists and to promote its future refinements, the REAM Foundation and Milken Institute's Center for Strategic Philanthropy have commissioned this commentary. The aim of this commentary is to independently review the methodology and outcome of the paper by Swedo et al. (2022). This commentary is structured into two main headings comprising: (1) discourse on the method, and (2) discourse on the results. Each section starts by discussing the benefits of the consensus followed by reviewing its limitations and offering directions for future work.

## Discourse on the method

Swedo et al. (2022) used a modified Delphi method to develop the consensus. This method has been used in healthcare for five decades (Gustafson et al., 1973). The main aim is to gather experts' opinions in a systematic manner in the absence of research evidence and/or widely agreed theoretical frameworks on a particular topic. Using the Delphi method, Swedo et al. (2022) structured the communication process among a group of experts and facilitated achieving a consensus definition for misophonia. However, because the consensus has been achieved it doesn't mean that the right definition for misophonia has been produced. Therefore, it is important to discuss strengths and weaknesses of the methods used as well as analyze the outcome in order to give directions to future work on this topic.

### Benefits

This is the first systematic project that achieved a consensus on a foundational definition for misophonia. As the study was mainly an internet-based inquiry, it is consistent with e-Delphi method (Donohoe et al., 2012). Use of e-Delphi methodology provided the researchers with several benefits comprising: (1) access to a wide range of experts from different locations, (2) cost-effectiveness, (3) good response and retention rates, and (4) offering equal opportunity to the committee members to express their opinions (Gordon and Pease, 2006). This e-Delphi involved a systematic review of the literature and several rounds of discussion and independent voting (*via* internet) by a committee of 15 invited experts and facilitated by the staff at Milken Institute. The committee included clinicians and researchers from different disciplines comprising audiology, neuroscience, psychology, neuropsychology, and psychiatry. Based on the review of literature the facilitators created a list of possible definitions for misophonia and its domains which went through the process of discussion and voting. “Consensus” was considered as 80% agreement of all committee members present when a given vote was conducted. The voting was anonymous which enabled the committee members to endorse the definition that they approved without problems associated with face-to-face disagreements. Using this systematic process by Swedo et al. (2022) was beneficial because it allowed for a group of experts with diverse professional background to examine the empirical studies on misophonia, discuss their agreements and disagreements and to achieve a consensus. They included input of all committee members which minimized some of the biases arising from unbalanced input of a few participants.

### Limitations

#### Literature search

The search strategy did not identify some of the studies on relevant topics (e.g., hyperacusis, noise sensitivity/noise annoyance, sensory processing disorders and autism spectrum disorder) in which some of the characteristics of misophonia in contexts of audiology, psychoacoustics, developmental psychology and environmental noise and health have been discussed (Bregman and Pearson, 1972; Guski et al., 1999; Aazh and Moore, 2017, 2018; Aazh et al., 2018; Danesh et al., 2021; Enzler et al., 2021). Reviewing such studies can improve our knowledge about possible underlying mechanisms of misophonia. Consistent with this, Brout (2022) challenged the overall literature review strategy in the consensus paper and highlighted its limitation as being too focused on observable behaviors and leaving out postulated mechanisms. She suggested that principles of Research Domain Criteria (RDoC) (Sanislow et al., 2010) should be used. RDoC promotes research that focuses on investigating the biological, physiological, and behavioral elements that comprise an illness rather than focusing on symptom-based diagnosis (Sanislow et al., 2010). It is worth mentioning that the members of the consensus committee were pioneers in misophonia research and have conducted many research studies consistent with RDoC principles. However, their conclusion was that as the underlying mechanism of misophonia is not fully understood, the inclusion of postulated mechanisms to the definition was not justified.

#### Committee members bias

One recognized limitation in Delphi method is it reliance on the opinion of experts on a topic. The individual experts can be sensitive about the topic, have conflicting theoretical positions, or be influenced by various professional or political agendas. Therefore, the data collected from experts may knowingly or unknowingly be biased (Turoff and Linstone, 2002).

#### Representativeness of the committee

The committee did not include non-professionals or individuals who themselves suffer from misophonia. Involvement of individuals with misophonia could challenge some of the concepts agreed by the professionals within the Delphi process and minimize the so called inbreeding of the viewpoints (Linstone, 2002).

#### Anonymity

The discussion round was not anonymous, and the committee members took part in a meeting *via* video for exchange of ideas. This can produce some limitations as the less confident members may be unwilling to participate in discussions. In other words, confidence, deference, and shyness could have influenced some of the conclusions drawn (McKenna, 1994). This can damage the integrity of the data (Chou, 2002). However, Swedo et al. (2022)'s study had 4 rounds of anonymous voting which is likely to have provided the committee members with plenty of opportunity to express their views.

#### Accountability

Although anonymity is an important principle in Delphi method, it can impact on accountability (McKenna, 1994). In the misophonia study, the rounds of voting were completely anonymous. Therefore, an individual committee member could have provided unjustified answers as their response will never be attributed to them. Accountability can be improved by asking follow-up questions in order to get clarification and justification on why a particular answer was given in a key round of votes.

## Discourse on the results

### Benefits

The e-Delphi study achieved consensus on describing misophonia and factors related to it. The work conducted by Swedo et al. (2022) is beneficial to healthcare professionals as it confers validity and specificity to misophonia. The result from the e-Delphi study (summarized in Table 1) is beneficial to patients too because it helps to understand their unique experience of misophonia, appreciate the nature of the problem that affects them and the relational effects on families and communities. Swedo et al. (2022)'s work takes us closer to the truth which subsequently can facilitate future treatments and support for this population.

**Table 1:** Summary of the consensus definition of misophonia.

**Domains**	**Consensus description**
General description	Misophonia is characterized by decreased tolerance to specific sounds or stimuli associated with such sounds (known as triggers). Exposure to triggers can evoke disproportionate emotional, physiological, and behavioral reactions leading to distress, and/or impairment in social, occupational, or academic functioning. Triggers often, but not exclusively, are stimuli generated by another human being's body.
Misophonic triggers	Most common triggers are auditory, but some may react to visual triggers too. Common triggers include but not limited to sounds associated with oral functions (e.g., chewing, eating, smacking lips, slurping, coughing, throat clearing, and swallowing.), nasal sounds (e.g., breathing and sniffing), non-oral/nasal sounds produced by people (e.g., pen clicking, keyboard typing, finger or foot tapping and shuffling footsteps), as well as sounds produced by objects (e.g., clock ticking) or sounds generated by animals. Examples of visual triggers are cracking knuckles and jiggling or swinging legs or watching someone eat.
Reactions to misophonic triggers	*Emotional:* Anger, irritation, disgust, and anxiety. *Physiological*: Increased muscular tension, increased heart rate, and sweating. *Behavioral reactions:* Agitation, aggression, avoidance, seeking to discontinue the triggering stimuli, and mimicking.
Influences on reactions	The strength of the reaction can be influenced by (1) the context, (2) the individual's perceived degree of control, and (3) the relationship with individual who is the source of the trigger.
Functional impairments	Impaired occupational and/or academic functioning, concentration difficulties, impaired social functioning, strained social relationships, and social isolation.
Relationship to other conditions/disorders	The symptoms of misophonia should not be better explained by any co-occurring disorders including but not limited to hearing impairment, tinnitus, hyperacusis, anxiety disorders, mood disorders, personality disorders, obsessive compulsive related disorders, post-traumatic stress disorder, autism spectrum disorder, and attention deficit hyperactivity disorder.

As shown in Table 1, the consensus produced one general description for misophonia and five domains comprising: (1) misophonic triggers, (2) reactions to misophonic triggers, (3) influences on reaction, (4) functional impairments, and (5) relationship to other conditions/disorders.

The general description of misophonia emphasizes that the main characteristic of misophonia is decreased tolerance to specific sounds leading to disproportionate reactions. The consensus covers a wide range of common misophonic triggers and the sufferer's reaction to them. They also elaborate that the individual's reaction is not always disproportionate, and it depends on the context and the source of the trigger. Swedo et al. (2022) described that misophonia can limit individuals' activities, restrict their participation in social and familial life, and impact on their psychological wellbeing. Finally, they agreed that other comorbid disorders with misophonia need to be investigated and treated when needed.

### Limitations and future directions

#### Misophonia: A “disorder” or a less common presentation of natural human variation?

Swedo et al. (2022) reported that there was disagreement among committee members on how to describe misophonia and only in Round 4 of the voting they reached an agreement that misophonia should be described as a “disorder.” However, the committee agreed that the scientific evidence regarding whether to classify misophonia as a “medical” or “psychiatric” disorder was insufficient. Although considering misophonia as a disorder is consistent with the negative experience of some of the sufferers (especially patients who seek help from professionals), it may not be true for everyone with misophonia. As the underlying mechanism of misophonia is not fully understood, classifying it as a disorder at this stage may be premature. Moreover, Naylor et al. (2021) conducted an online survey completed by medical students at University of Nottingham in the UK. They found that 49.1% of 336 respondents had misophonia as measured by the Amsterdam Misophonia Scale (A-MISO-S) (Schroder et al., 2013). Although this high prevalence may be as the result of their survey method or characteristics of their participants, it would be unreasonable to suggest that about 50% of this sample had a “disorder.” It is possible that misophonia has a spectrum and on the extreme end it can be a debilitating disorder and on the other end it may represent a less common presentation of natural human variation. This is consistent with the concept of neurodiversity (Blume, 1998). According to neurodiversity principles, classifying misophonia as a disorder can be stigmatizing and results in a focus on dysfunctions and impairments as opposed to focusing on understanding the differences between people (Kapp et al., 2013). Instead, misophonia can be described as an “experience”. This shifts the balance from considering it as a “disorder” that is dysfunctional and should be cured to promoting accommodation/adjustment in work place, school, and other public places in order to improve quality of life for people who experience misophonia (Porcaro et al., 2019). To sum up, more research is needed to explore misophonia phenomenon and to understand if it is indeed a disorder. In the meantime, clinicians can benefit from the consensus definition (Table 1) as a guide that highlights factors that they need to explore with their patients in order to fully understand their experience of misophonia and to appreciate the nature of the problems faced by them, its relational effect on their families and to formulate individualized treatment plan and care.

#### Distinguishing misophonia from hyperacusis and noise sensitivity

It can be important to differentiate misophonia from other types of sound intolerance (e.g., hyperacusis and noise sensitivity/noise annoyance). Hyperacusis is defined as an intolerance of certain everyday sounds, which are perceived as too loud or uncomfortable and which cause significant distress and impairment in the individual's day-to-day activities (Aazh et al., 2022b). For an individual with hyperacusis, certain everyday sounds, such as kitchen noises, bangs, music, slamming doors, water running, cutlery on plates, traffic noise, hair dryers, and hand dryers, are perceived as too loud, uncomfortable, or painful. Noise sensitivity is a personality trait about attitudes toward noise in general (Baliatsas et al., 2016). A person with high noise sensitivity may perceive noise caused by neighbors, nearby factories, workshops, farms, radiators, air conditioning, and background music as disruptive and distressing (Weinstein, 1978). Future definitions for misophonia may include statements excluding the possibility of hyperacusis and/or noise sensitivity. For example, to exclude hyperacusis, this statement can be used: “The person's primary reaction to sounds is not aural pain or physical discomfort in their ears due to excessive loudness of the sounds.” To exclude noise sensitivity, this statement can be used: “The person's sound intolerance is not better explained by their general attitude toward noise and environmental noise pollution (e.g., noise from neighbors, nearby airports, traffic, workshops, internal plumbing, or air conditioning).” These are just examples and adoption of such statements needs to be supported by empirical evidence.

#### How to establish if symptoms of misophonia are better explained by any co-occurring disorders?

According to the consensus definition, misophonia is present if its symptoms are not better explained by something else. In other words, an individual who exhibits a disproportionate emotional, physiological, or behavioral reaction when exposed to misophonic triggers may not necessarily have misophonia. Their symptoms may be better explained by a co-occurring disorder. In this section, the focus is to discuss how symptoms of co-occurring disorders (excluding hyperacusis and noise sensitivity which have already been discussed in the previous section) can be misinterpreted as misophonia. To make this matter clearer several examples are given here: (1) Schroder et al. (2013) explained that although an individual might feel distressed when exposed to certain oral/nasal triggers, their reaction may be related to their general obsession about contamination and to an underlying anxiety disorder, as opposed to misophonia. (2) Cavanna and Seri (2015) reported that in certain individuals, behavioral reactions to misophonic triggers such as mimicking or reproducing the triggers can be related to an underlying tic disorder as opposed to misophonia. (3) In the Third International Conference on Hyperacusis, Hashir Aazh discussed a case study of a patient whose main reason for feeling anxious and angry when exposed to the trigger sounds was her magical thinking (Dubal and Viaud-Delmon, 2008), visual hallucinations and an underlying psychosis as opposed to misophonia or hyperacusis (Aazh et al., 2018). (4) Some individuals with tinnitus might exhibit adverse reaction to certain misophonic triggers, because they may be afraid that certain sounds can make their tinnitus worse (Aazh et al., 2022a). Therefore, reaction to misophonia triggers is because of their tinnitus as opposed to misophonia.

It is important to highlight that although misophonia can coexist with other disorders, the possibility that misophonia symptoms are not related to misophonia but to a co-occurring condition is very rare (based on the author's clinical experience).

## Conclusions

The method used by Swedo et al. (2022) for the misophonia definition was an established method in health and social care to gather experts' opinions and to achieve a consensus in the absence of complete empirical evidence or widely agreed theories. The combination of the reliable techniques and the systematic process utilized in this e-Delphi is likely to have minimized the risk for bias as much as it was possible. However, consensus is a viewpoint (Vernon, 2009), therefore it should not be misinterpreted as literal fact. To further improve representativeness of the committee, future studies should involve patients with a variety of misophonia symptoms and severity. It may be premature to consider misophonia as a disorder given the lack of empirical evidence regarding any possible underlying pathology on one hand and due to its seemingly high prevalence among the population on the other. Future definitions can use certain criteria to distinguish misophonia from hyperacusis and noise sensitivity. It is possible that in some individuals, misophonia symptoms are better explained by a co-occurring disorder, but this is very rare.

## Author contributions

The author confirms being the sole contributor of this work and has approved it for publication.
